# The Role of Metabolites in CSF on NAFLD Development: A Mendelian Randomisation Analysis

**DOI:** 10.1002/edm2.70088

**Published:** 2025-11-23

**Authors:** Fang Liang, Tiegang Xiao, Bing Wang

**Affiliations:** ^1^ Department of Traditional Chinese Medicine Shanghai Sixth People's Hospital Affiliated to Shanghai Jiao Tong University School of Medicine Shanghai China

**Keywords:** causal relationship, cerebrospinal fluid metabolites, genome‐wide association study, Mendelian randomisation, non‐alcoholic fatty liver disease

## Abstract

**Background:**

Non‐alcoholic fatty liver disease (NAFLD) is a prevalent chronic liver condition linked to metabolic syndrome. Recent studies suggest that metabolites in cerebrospinal fluid (CSF) may play a role in NAFLD development; however, the specific causal relationship between the two remains unclear.

**Methods:**

This bidirectional two‐sample Mendelian randomisation (MR) study analysed the causal relationships between 337 CSF metabolites and NAFLD. We used genome‐wide association study (GWAS) datasets from the OpenGWAS and FinnGen databases. MR analysis was mainly conducted through the inverse‐variance weighted method from the ‘TwoSampleMR’ R package, and sensitivity analyses were conducted to validate findings.

**Results:**

A total of 24 CSF metabolites were found to have significant causal relationships with NAFLD, with 13 identified in the discovery group and 13 in the validation group. These metabolites predominantly include amino acids, amino acid derivatives, esters, lipids, lipid derivatives, nucleotides, organic acids, carbohydrates and vitamins. Notably, metabolites of lipid derivatives such as 7‐alpha‐hydroxy‐3‐oxo‐4‐cholestenoate (7‐HOCA) exhibited a consistent positive causal effect on NAFLD, and nucleotide metabolites uracil showed a consistent inverse causal effect on NAFLD both in the discovery and validation groups. Sensitivity analyses showed robust results without significant pleiotropy or heterogeneity.

**Conclusion:**

This study reveals significant causal associations between specific CSF metabolites and NAFLD, emphasising the importance of the brain‐liver axis in NAFLD pathogenesis. These findings provide a scientific basis for developing early diagnostic biomarkers and personalised therapeutic strategies targeting CSF metabolites, potentially improving NAFLD management and patient outcomes.

## Graphical abstract

This MR analysis reveals 24 CSF metabolites, notably 7‐HOCA (positively correlated) and uracil (negatively correlated), as causal factors in NAFLD pathogenesis. These findings implicate CNS derived metabolic regulation in hepatic steatosis, proposing novel diagnostic biomarkers and therapeutic targets. The robust genetic evidence underscores the brain‐liver axis' role in metabolic disease progression, with potential clinical applications in risk stratification and precision medicine approaches for NAFLD.

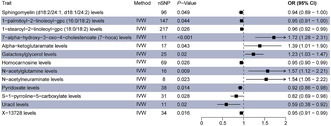

**Abbreviations**
7‐HOCA: 7‐alpha‐hydroxy‐3‐oxo‐4‐cholestenoate
CNS: central nervous system
CSF: cerebrospinal fluid
GWAS: genome‐wide association study
IVs: instrumental variables
IVW: inverse variance weighted
MR: Mendelian randomisation
NAFLD: non‐alcoholic fatty liver disease
SNPs: single nucleotide polymorphisms

## 1 Introduction

With the global rise in obesity rates, non‐alcoholic fatty liver disease (NAFLD) has become one of the most prevalent chronic liver diseases, affecting approximately 25% of the adult population worldwide [1, 2, 3]. NAFLD includes a spectrum of conditions ranging from simple steatosis to non‐alcoholic steatohepatitis (NASH) and can progress to cirrhosis and liver cancer [4, 5, 6]. Although the exact mechanisms underlying NAFLD are not fully understood, its strong association with metabolic syndrome suggests that metabolic abnormalities play a crucial role in its pathogenesis [7, 8, 9].

The central nervous system (CNS) plays a vital role in liver health by regulating appetite, energy expenditure and metabolic balance [10, 11]. Cerebrospinal fluid (CSF), a primary component of the CNS, mirrors the biochemical activity of the brain and its surrounding tissues, offering a unique perspective for investigating the connection between the brain and systemic diseases [12, 13]. While recent research on NAFLD has mainly concentrated on peripheral blood markers, the relationship between CSF metabolite changes and NAFLD remains underexplored. CSF metabolites, such as uracil and 7‐alpha‐hydroxy‐3‐oxo‐4‐cholestenoate (7‐HOCA) [14, 15, 16], may be directly involved in the development of NAFLD through biochemical pathways linked to lipid metabolism, inflammatory response and energy balance. Uracil, a degradation product of pyrimidine nucleotides, is commonly used as a marker of nucleic acid metabolism [17, 18, 19]. Elevated levels of uracil may indicate increased cellular nucleic acid metabolism, potentially linked to liver metabolic health [20, 21, 22]. Conversely, 7‐HOCA, an intermediate in the bile acid synthesis pathway, may signal bile acid metabolic dysregulation when elevated, which is closely related to the progression of NAFLD [23, 24]. Consequently, investigating the causal relationship between CSF metabolites and NAFLD is crucial for understanding the pathogenesis of NAFLD, discovering new diagnostic markers and developing effective therapeutic strategies.

Mendelian randomisation (MR) is a powerful methodology for investigating the causal relationships between exposure and outcome variables [25]. Unlike traditional observational studies, MR utilises genetic variations as instrumental variables (IVs) to effectively mitigate confounding factors and probe the molecular mechanisms connecting exposure factors to disease outcomes [26]. This study aims to leverage genome‐wide association study (GWAS) datasets from public databases and employ MR analysis to explore the causal relationship between different levels of CSF metabolites and NAFLD. Our objective is to reveal the intrinsic connections between CNS metabolic processes and systemic metabolic diseases, identifying new biomarkers and potential therapeutic targets for the prevention and treatment of NAFLD and other metabolic disorders. Additionally, this study will investigate the direct association between brain metabolic activity and liver health, potentially challenging the traditional focus on peripheral biomarkers and offering valuable insights into the complex interactions between the brain and systemic metabolic states. The results of this research could not only enhance the understanding of NAFLD pathogenesis but also provide a new theoretical basis for broader metabolic disease research.

## 2 Materials and Methods

### 2.1 Data Sources

This study is a bidirectional two‐sample MR study following the STROBE‐MR guidelines (Strengthening the Reporting of Mendelian Randomisation Studies) [27]. We utilised data on 337 CSF metabolite levels from the OpenGWAS database (https://gwas.mrcieu.ac.uk/) as exposure variables and NAFLD as the outcome for the forward MR analysis. Based on the original authors' methodological description [28], the genetic instruments for CSF metabolites were derived from a meta‐analysis of two cohort studies (WADRC and WRAP) with a combined sample size of 291 participants (155 from WADRC and 136 from WRAP). Metabolite levels were quantified using ultrahigh performance liquid chromatography–tandem mass spectrometry (UPLC‐MS/MS) platform by Metabolon Inc. The GWAS analyses employed linear regression models with an additive genetic effect, adjusting for age at CSF collection, sex, the first five principal components and genotyping batch. To ensure the reliability of the results, we selected NAFLD‐related GWAS data from different databases for the forward analysis. Specifically, NAFLD data from the OpenGWAS database (ebi‐a‐GCST90091033, cases: 8434, controls: 770,180) were used for the discovery group [29], and NAFLD data from the FinnGen database (finngen_R10_NAFLD, cases: 2568, controls: 409,613) were used for the validation group [30]. In the reverse analysis, the outcome variables from the forward analysis were used as exposures, and the significant metabolites from the forward analysis were used as outcomes. Independent two‐sample MR analyses were conducted separately for both the discovery and validation groups. Data were selected to ensure they came from European populations (CSF metabolites data were derived from USA, while the genetic data for NAFLD were primarily from UK and Finnish populations, there is no sample overlap), with a preference for datasets with the largest sample sizes or the highest number of cases. Through this approach, we aim to reveal the causal relationships between NAFLD and specific CSF metabolites, providing potential biomarkers for the prevention and treatment of NAFLD.

### 2.2 IVs Selection

#### 2.2.1 Selection of Exposure‐Related IVs

We employed the ‘TwoSampleMR’ R package to perform the two‐sample MR analysis [31], identifying single nucleotide polymorphisms (SNPs) strongly associated with the exposure factors as IVs. The parameters set for IV selection included a *p*‐value threshold of 5 × 10^−6^, a linkage disequilibrium (LD) threshold of 0.001, and a window size of 10,000 kilobases [25, 32, 33]. The same *p*‐value threshold was used for both the forward and reverse analyses. The IVs selected had to meet the three core assumptions of MR analysis [26, 34]: relevance (SNPs must be strongly associated with the exposure), independence (SNPs must be independent of confounders) and exclusion restriction (SNPs must influence the outcome only through the exposure). For all IVs, we ensured complete data availability by supplementing missing sample size information from databases such as the OpenGWAS database.

#### 2.2.2 Removing Confounding Factors

To remove potential confounding IVs, we used the LDtrait tool from the LDlink database (https://ldlink.nih.gov/?tab=ldtrait) [35, 36]. This tool evaluates the associations between specific SNPs and various phenotypes. In the forward analysis, to minimise the risk of horizontal pleiotropy, we excluded IVs that were significantly associated with potential confounding conditions (*p* < 5 × 10^−6^) including chronic viral hepatitis (B and C), alcoholic liver disease, autoimmune hepatitis, primary biliary cholangitis, hemochromatosis and Wilson's disease. We also excluded IVs associated with known risk factors for NAFLD including obesity, type 2 diabetes and dyslipidemia at the same significance threshold. In the reverse analysis, IVs associated with CNS‐related phenotypes (*p* < 5 × 10^−6^) including Alzheimer's disease, Parkinson's disease, multiple sclerosis, epilepsy, depression, schizophrenia and stroke or the metabolites themselves were similarly excluded as confounders. Additionally, in both forward and reverse analyses, we excluded IVs associated with the outcome at a threshold of *p* < 5 × 10^−6^ to further mitigate the risk of horizontal pleiotropy and strengthen the validity of our causal estimates.

After excluding these potential confounding IVs, we calculated the *F*‐statistic (*F* = beta^2^/se^2^) for the remaining IVs, ensuring all had *F*‐statistics > 10 to maintain the statistical power and strength of the MR analysis [37, 38, 39]. We then matched the effect alleles and reference alleles for the IVs related to both exposures and outcomes and used the ‘MR‐PRESSO’ R package to identify and exclude outlier IVs [40, 41]. This data cleaning step resulted in the final set of IVs for the MR analysis.

### 2.3 MR Analysis

The MR analysis was primarily conducted using the ‘TwoSampleMR’ R package [42], designed for two‐sample MR analyses. To ensure accuracy, independent MR analyses were performed for both the discovery and validation groups to evaluate the causal relationships between variables from different data sources. If only one SNP was available for a particular analysis, the Wald ratio method was used [26, 43]. When more than two SNPs were available, the inverse variance weighted (IVW) method (random effect model) was primarily used [44, 45], supplemented by MR Egger [46], weighted median [47], simple mode [48] and weighted mode [49] methods for further verification. Significant results were based on IVW method *p*‐values < 0.05, and consistency in beta values across additional methods [25, 50]. Given the exploratory nature of our current analysis, we primarily report findings with raw *p*‐values < 0.05, while also implementing Bonferroni correction for multiple testing and report results that remained significant after Bonferroni adjustment (adjusted *p* < 0.05) as supplementary evidence. Using the ‘TwoSampleMR’ R package, we calculated the odds ratios (OR) and 95% confidence intervals (CI) for each analysis direction to assess the causal effects revealed by the MR analysis.

### 2.4 Sensitivity Analysis

The main purpose of sensitivity analysis was to explore whether the IVs were influenced by horizontal pleiotropy and heterogeneity [51]. When more than two SNPs were involved, the *Cochrane's Q statistic* was used to assess heterogeneity [52]. A *p*‐value of *Cochrane's Q statistic* < 0.05 indicated significant heterogeneity, suggesting potential violations of MR assumptions or the presence of distinct causal pathways through which different genetic variants might influence the outcome [53, 54]. To detect horizontal pleiotropy, the MR Egger intercept method was used when there were more than two SNPs, and the MR‐PRESSO global test was applied if there were more than four SNPs [41, 55]. We also visualised the sensitivity analysis results using scatter plots and funnel plots, which helped reveal the data distribution and potential biases. Additionally, we conducted a leave‐one‐out analysis by sequentially removing each IV and recalculating the MR results to assess the contribution of each IV to the overall results. These methods ensured the robustness and reliability of our study, enhancing our understanding of the relationship between CSF metabolite levels and NAFLD and providing critical scientific support for precision medicine.

### 2.5 Reverse MR Analysis

The reverse analysis evaluated the causal relationships between NAFLD from different databases and the significant CSF metabolites identified in the forward analysis (CSF metabolite levels to NAFLD). The parameters for IV selection in this analysis were consistent with those used in the forward analysis. If an IV was significantly associated with outcome‐related phenotypes, including but not limited to peripheral blood metabolite levels, other CSF metabolite levels, liver function markers and metabolic traits, it was identified as a potential confounder and excluded. The number of SNPs identified for each metabolite in the reverse analysis is detailed in Tables S6 and S7 for the discovery and validation groups, respectively. The remaining methods for the reverse MR analysis were consistent with those used in the forward MR analysis.

## 3 Results

### 3.1 Causal Effects of Different CSF Metabolite Levels on NAFLD

Following MR guidelines, we first selected SNPs closely associated with the exposure variables (337 CSF metabolite levels). After excluding potential confounders and filtering for *F*‐statistics, we obtained 11,174 SNPs for the discovery group (337 CSF metabolite levels to NAFLD (ebi‐a‐GCST90091033)). Detailed information about these SNPs is provided in Table S1. For the validation group (337 CSF metabolite levels to NAFLD (finngen_R10_NAFLD)), 14,730 SNPs were obtained, with details in Table S2. All SNPs had *F*‐statistics > 10, ensuring the reliability of the IVs included in the analysis.

**FIGURE 1:**
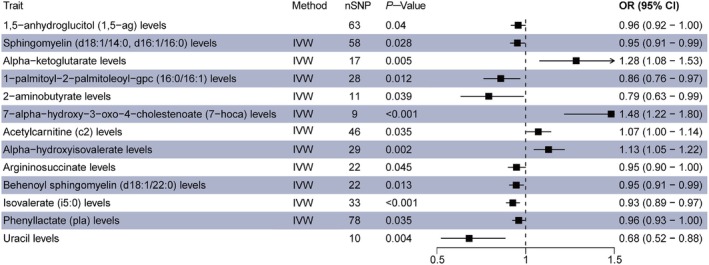
Forest plot shows the significant results of MR analysis in discovery group. CI, confidence interval; IVW, inverse variance‐weighted; MR, Mendelian randomisation; nSNP, number of single nucleotide polymorphism; OR, odds ratio.

Using the IVW method in the primary MR analysis, we observed significant causal relationships between 13 CSF metabolites and NAFLD in the discovery group (Figure 1). Specifically, levels of acetylcarnitine (C2), alpha‐ketoglutarate, alpha‐hydroxyisovalerate, and 7‐HOCA showed positive causal effects on NAFLD. In contrast, levels of 2‐aminobutyrate, 1‐palmitoyl‐2‐palmitoleoyl‐GPC (16:0/16:1), phenyllactate, uracil, sphingomyelin (d18:1/14:0, d16:1/16:0), argininosuccinate, behenoyl sphingomyelin (d18:1/22:0), 1,5‐anhydroglucitol (1,5‐AG) and isovalerate (I5:0) showed negative causal effects. Notably, 7‐HOCA (adj‐*p* = 0.027) and isovalerate (I5:0) (adj‐*p* = 0.043) also demonstrated significant associations even after Bonferroni correction. Detailed MR analysis results for the discovery group are provided in Table S3.

**FIGURE 2:**
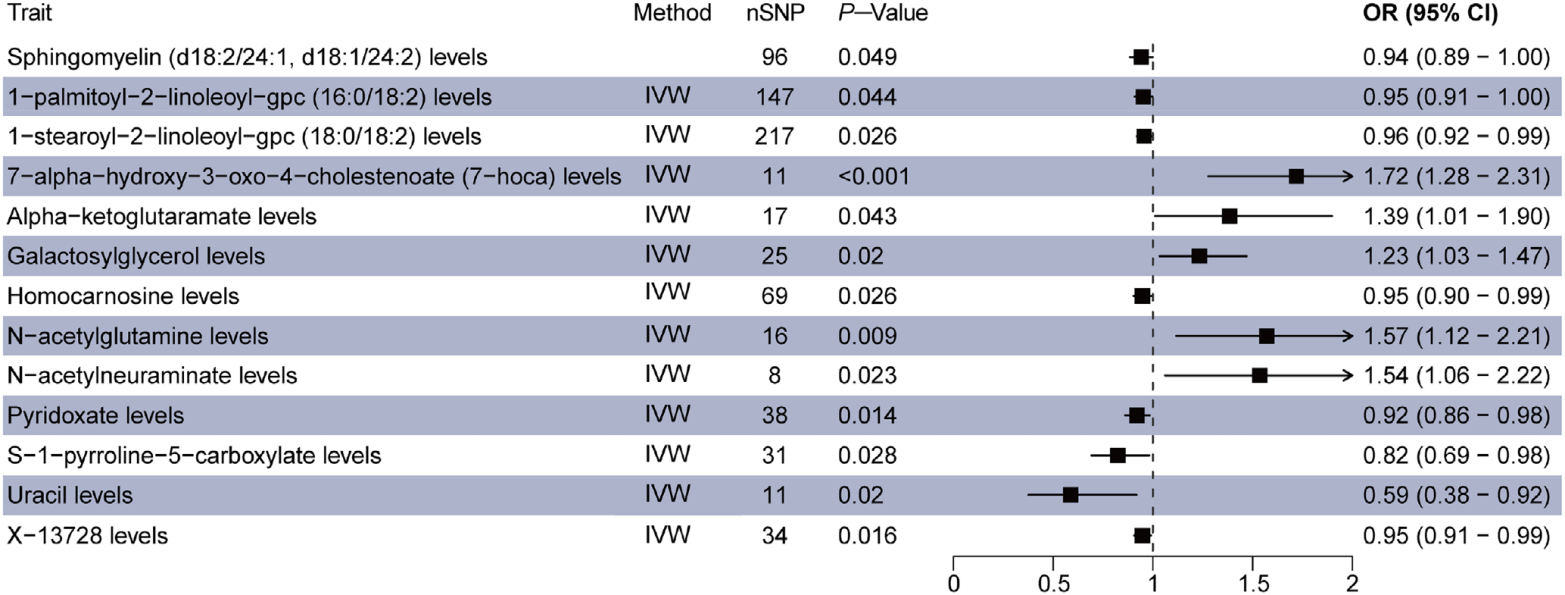
Forest plot shows the significant results of MR analysis in the validation group. CI, confidence interval; IVW, inverse variance‐weighted; MR, Mendelian randomisation; nSNP, number of single nucleotide polymorphism; OR, odds ratio.

The MR analysis results for the validation group (Figure 2) also identified significant causal associations between 13 CSF metabolites and NAFLD. Positive causal effects were observed for pyridoxate, 1‐stearoyl‐2‐linoleoyl‐GPC (18:0/18:2), alpha‐ketoglutarate, N‐acetylglutamine, galactosylglycerol, and 7‐HOCA. Negative causal effects were found for S‐1‐pyrroline‐5‐carboxylate, sphingomyelin (d18:2/24:1, d18:1/24:2), N‐acetylneuraminate, 1‐palmitoyl‐2‐linoleoyl‐GPC (16:0/18:2), X‐13728, uracil and homocarnosine. In the validation cohort analysis, no results remained significant after multiple testing correction (detailed MR analysis results provided in Table S4). Despite this overall lack of significance in the validation cohort, it is noteworthy that 7‐HOCA and Uracil levels demonstrated significant causal relationships with NAFLD in both the discovery and validation groups, regardless of the direction of effect.

### 3.2 Sensitivity Analysis

In the forward analysis, no significant pleiotropy or heterogeneity was observed in any direction (Table S5). For the 13 significant MR analysis directions in the discovery group (Figure S1A–M) and the 13 significant MR analysis directions in the validation group (Figure S2A–M), the causal effect estimates of NAFLD associated with individual SNPs showed marked variability. However, when we performed leave‐one‐out analysis by sequentially removing each SNP from the analysis direction, the results (Figure S3A–M for the discovery group and Figure S4A–M for the validation group) showed that removing a single SNP only affected the range of maximum and minimum causal effect estimates, with minimal impact on the overall direction of the causal effect estimate. This suggests that, despite the variability in the causal effects of individual SNPs in different analysis directions, the overall causal effect estimates remain consistent.

**FIGURE 3:**
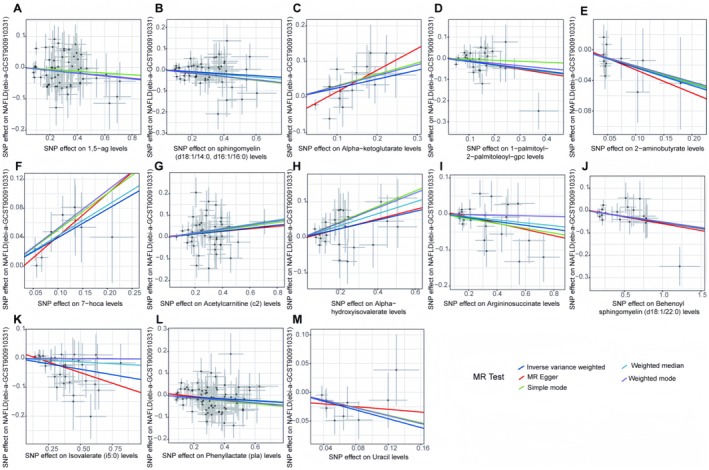
Scatter plots show the causal effects of cerebrospinal fluid metabolites on non‐alcoholic fatty liver disease from the OpenGWAS database. The dots represent the instrumental variables included in the current analysis; the lines represent the trends fitted by the causal effects obtained by different Mendelian randomisation analysis methods, and the colours of the lines represent different Mendelian randomisation methods, as shown in the legend.

**FIGURE 4:**
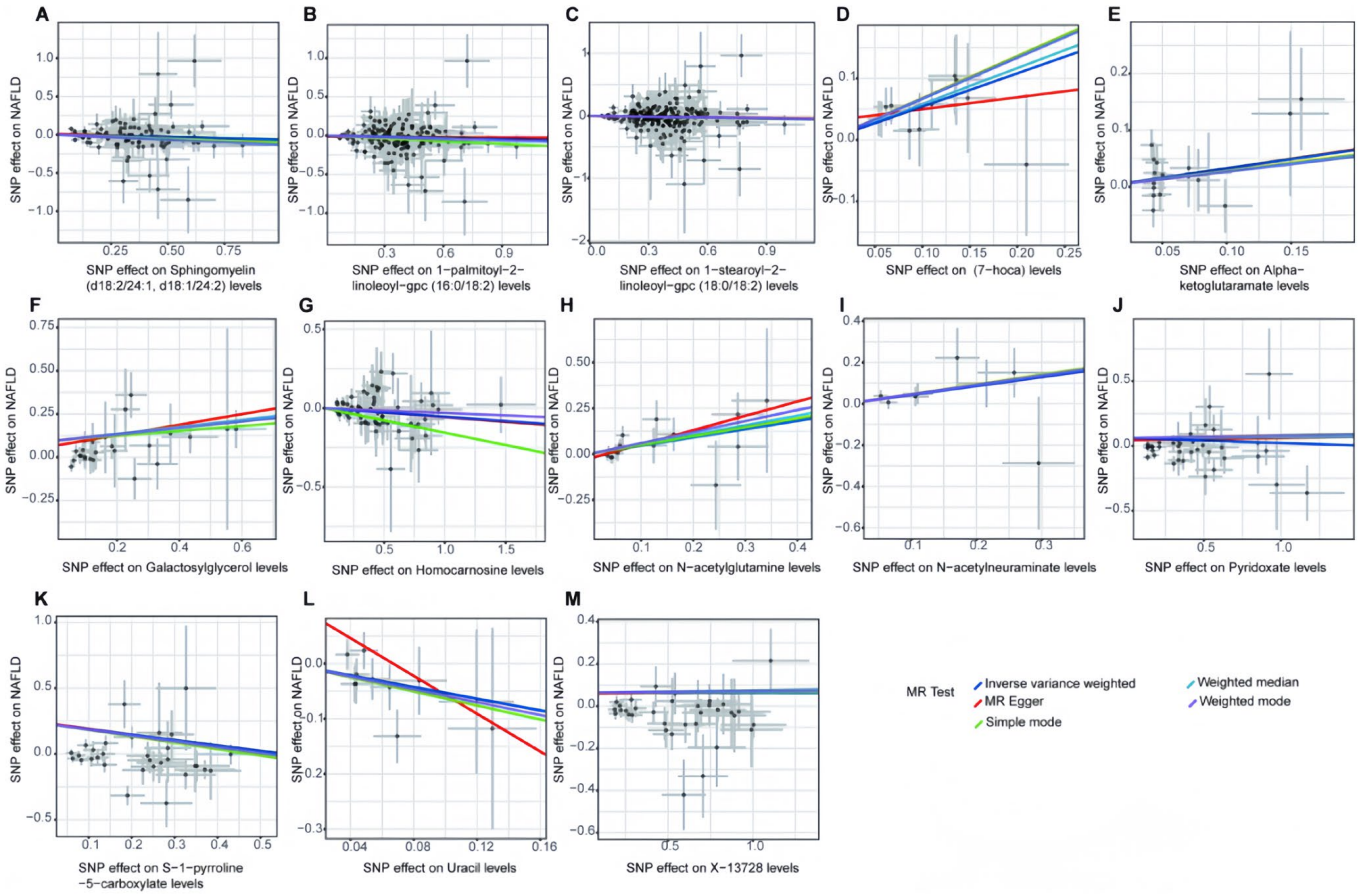
Scatter plots shows the causal effects of cerebrospinal fluid metabolites on non‐alcoholic fatty liver disease from FinnGen database. The dots represent the instrumental variables included in the current analysis, the lines represent the trends fitted by the causal effects obtained by different Mendelian randomisation analysis methods, and the colours of the lines represent different Mendelian randomisation methods, as shown in the legend.

**FIGURE 5:**
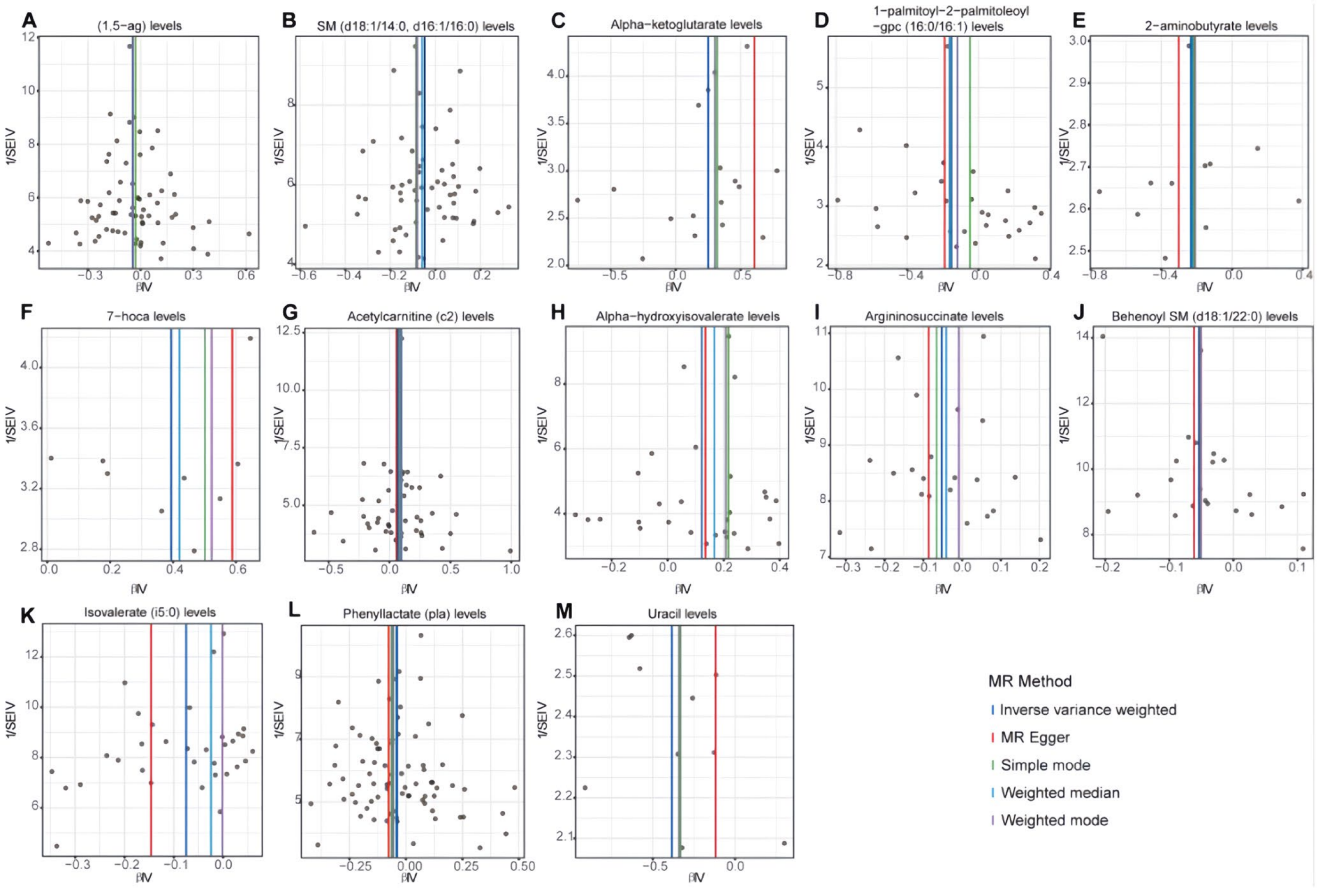
Funnel plots show the distribution of instrumental variables included in the analysis directions of the discovery group. The dots represent the instrumental variables included in the current analysis, the lines represent the trends fitted by the causal effects obtained by different Mendelian randomisation analysis methods, and the colours of the lines represent different Mendelian randomisation methods, as shown in the legend.

**FIGURE 6:**
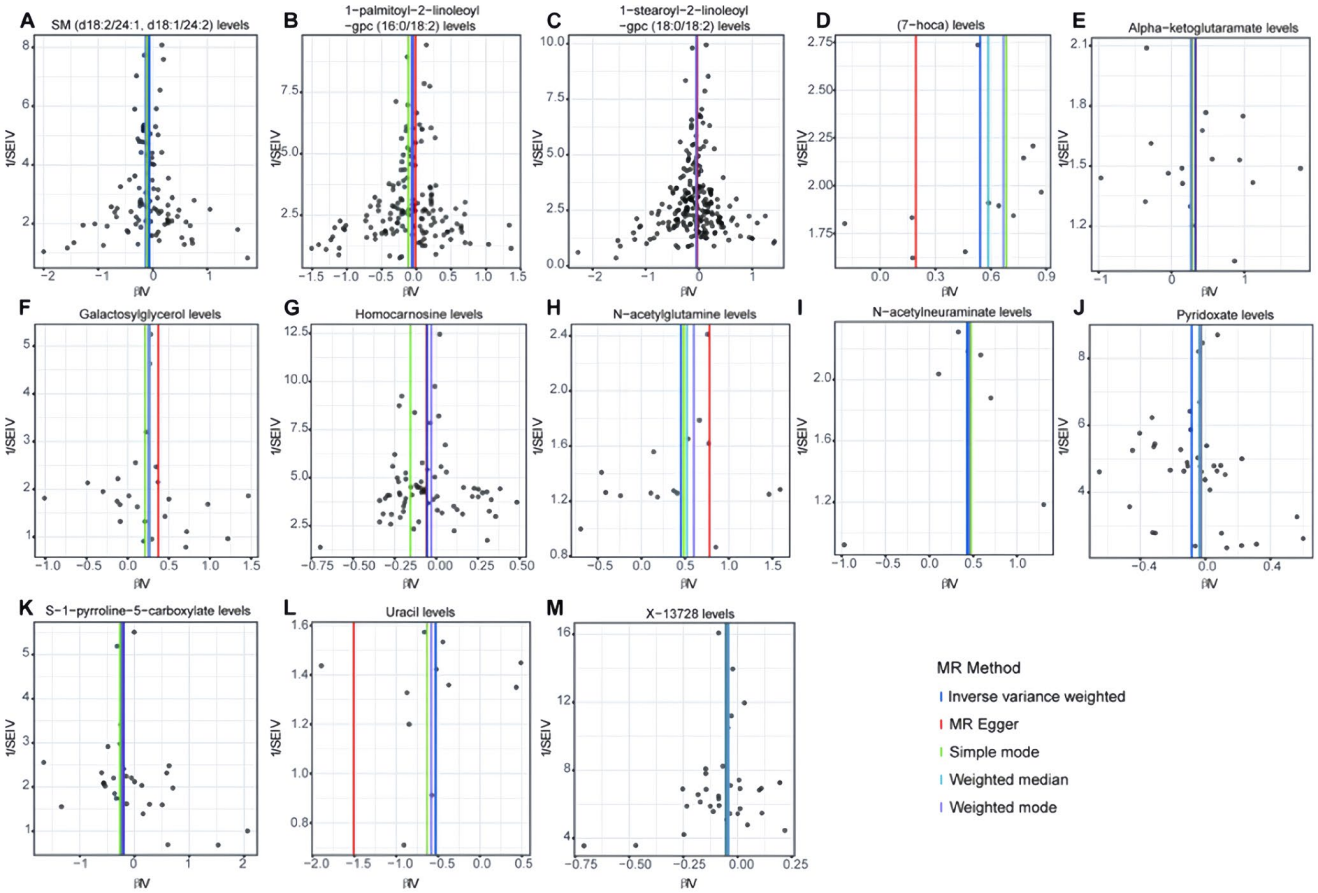
Funnel plots show the distribution of instrumental variables included in the analysis directions of the validation group. The dots represent the instrumental variables included in the current analysis, the lines represent the trends fitted by the causal effects obtained by different Mendelian randomisation analysis methods, and the colours of the lines represent different Mende.

Scatter plots for the forward analysis (Figure 3A–M for the discovery group and Figure 4A–M for the validation group) display the effect values of SNPs included in the analysis for the exposure and outcome. The distribution of data points around the regression line is consistent with our MR analysis results, indicating the identified causal relationships between exposure and outcome in various analysis directions. Funnel plots (Figure 5A–M for the discovery group and Figure 6A–M for the validation group) also showed no significant distribution anomalies for SNPs under the IVW method, further confirming the robustness of the analysis.

### 3.3 Reverse MR Analysis

The reverse MR analysis investigated the causal relationships between NAFLD from different databases and the significant CSF metabolite levels identified in the forward analysis. In the discovery group, we identified 11 SNPs significantly associated with NAFLD, with detailed information on their corresponding significant CSF metabolites from the forward analysis presented in Table S6. Similarly, in the validation group, 14 SNPs significantly associated with NAFLD were identified, with their corresponding significant CSF metabolite details shown in Table S7. However, despite these identified genetic instruments, no significant causal relationships were found between NAFLD and the aforementioned CSF metabolite levels in any direction (Table S8 for the discovery group and Table S9 for the validation group).

## 4 Discussion

This study employed the MR method to uncover causal relationships between specific CSF metabolites and NAFLD. Overall, we found that levels of amino acid metabolites (2‐aminobutyrate, Argininosuccinate, S‐1‐pyrroline‐5‐carboxylate) exhibited negative causal relationships with NAFLD, whereas most amino acid derivative metabolites (except Homocarnosine, which also had a negative causal relationship with NAFLD), such as alpha‐ketoglutarate, alpha‐hydroxyisovalerate, alpha‐ketoglutaramate, and N‐acetylglutamine, showed positive causal relationships with NAFLD. Carbohydrates (1,5‐anhydroglucitol) and carbohydrate derivatives (Galactosylglycerol, N‐acetylneuraminate) showed inconsistent causal effects on NAFLD between the discovery and validation groups. Additionally, only one ester metabolite (Acetylcarnitine) had a positive causal effect on NAFLD in the discovery group. Among lipid metabolites, several phosphatidylcholines (1‐palmitoyl‐2‐palmitoleoyl‐GPC, 1‐palmitoyl‐2‐linoleoyl‐GPC) and sphingomyelins (d18:1/14:0, d16:1/16:0; d18:1/22:0; d18:2/24:1, d18:1/24:2) exhibited protective effects against NAFLD, while 1‐stearoyl‐2‐linoleoyl‐GPC showed a positive causal relationship with NAFLD risk. The lipid derivative 7‐HOCA and vitamin metabolite pyridoxate also demonstrated positive causal effects on NAFLD. Furthermore, nucleotide metabolites (Uracil) and organic acid metabolites (Phenyllactate, Isovalerate) had negative causal relationships with NAFLD. The reverse MR analysis indicated that NAFLD did not significantly influence these metabolite levels, suggesting a potential unidirectional relationship from metabolites to disease risk. Sensitivity analyses showed no significant pleiotropy in the MR results, and leave‐one‐out analysis indicated that removing individual SNPs had minimal impact on the overall causal effect estimates, supporting the methodological robustness of our findings. These findings suggest potential metabolic connections between the CNS and liver pathophysiology in NAFLD development. The differential effects observed across metabolite classes—with certain amino acids and sphingomyelins showing protective associations while specific amino acid derivatives and lipid metabolites demonstrated risk‐promoting effects—indicate that CSF metabolite profiles may reflect broader systemic metabolic dysregulation in NAFLD. While the biological mechanisms underlying these relationships require further elucidation, our results contribute valuable insights into the complex metabolic networks that may influence NAFLD pathogenesis. The consistency of certain findings across discovery and validation cohorts, particularly for 7‐HOCA and Uracil, strengthens confidence in these associations. Future research should focus on understanding the mechanistic pathways connecting these metabolites to hepatic metabolism and exploring their potential utility in NAFLD risk assessment, while acknowledging that translation to clinical applications will require comprehensive validation studies.

From a clinical perspective, the present analysis has the following translational values: First, improving early diagnosis and risk assessment. NAFLD lacks specific clinical manifestations, making early diagnosis challenging. However, given its potential progression to cirrhosis and hepatocellular carcinoma, early detection and intervention are crucial for optimal patient outcomes [56, 57]. The CSF metabolites causally linked to NAFLD in our study could serve as novel biomarkers for early disease detection. Specifically, 7‐HOCA, which demonstrated a positive causal effect on NAFLD development, reflects alterations in lipid metabolism that may precede clinical liver dysfunction [58, 59]. By incorporating these metabolite profiles into clinical assessment, healthcare providers could not only identify high‐risk individuals before conventional liver damage markers appear, but also stratify patients according to their progression risk, enabling personalised preventive interventions.

Second, informing personalised treatment strategies. By establishing causal relationships between specific CSF metabolites and NAFLD, our findings may contribute to understanding metabolic pathways relevant to therapeutic interventions. For instance, while nutritional approaches have long been established as effective treatments for NAFLD [60, 61], our identification of 7‐HOCA's causal relationship with NAFLD provides additional insights into specific metabolic pathways that could be considered in treatment planning. Similarly, the protective effect of Uracil against NAFLD suggests that pyrimidine metabolism pathways warrant further investigation for their potential hepatoprotective mechanisms. These metabolite‐specific findings may help refine existing therapeutic approaches by providing a better understanding of the underlying metabolic processes; though clinical validation of these pathways will be necessary before implementation in personalised treatment strategies.

Third, understanding metabolic mechanisms. The identified CSF metabolites may provide insights into the systemic metabolic alterations associated with NAFLD progression. While CSF sampling is not practical for routine clinical monitoring due to its invasive nature and associated costs, these findings contribute to our understanding of metabolic pathways that may be reflected in more accessible biomarkers. Future research could explore whether similar metabolic patterns are detectable in blood or other easily obtainable samples, potentially leading to the development of less invasive monitoring approaches.

Current analysis not only established associations between CSF metabolites and NAFLD but also revealed the genetic foundation underlying these relationships. The SNPs we identified as IVs represent more than statistical tools; they reflect specific regulatory mechanisms of metabolic pathways. For Uracil‐related SNPs, we identified 10 genetic variants (rs10014125, rs10829493, rs11605070, rs117738218, rs117738726, rs2964955, rs4698735, rs6545320, rs6856183, rs78510655) primarily distributed near genes involved in pyrimidine metabolism. Notably, rs117738726 is proximal to the DYDC1, DYDC2 and MAT1A gene cluster, which participates in methionine metabolism and methylation processes, potentially regulating pyrimidine synthesis through epigenetic mechanisms [62, 63]. The rs4698735 locus is adjacent to BOD1L1 and NKX3‐2 genes, which are involved in DNA repair and transcriptional regulation, affecting nucleic acid metabolic balance [64, 65, 66]. Additionally, rs78510655 is near GPR68 and CCDC88C, suggesting that G protein‐coupled receptor signalling pathways may play a role in Uracil metabolism regulation [67, 68, 69]. The negative correlation between Uracil and NAFLD may derive from these SNPs influencing AMPK signalling pathways and mitochondrial function, thereby regulating hepatic fatty acid oxidation and energy homeostasis.

For 7‐HOCA‐related SNPs (rs10128172, rs10133012, rs117154811, rs118123321, rs4628111, rs632005, rs75165358, rs77511096, rs79290247), rs10128172 is located near the ZMIZ1 gene, which encodes a transcriptional co‐activator involved in steroid hormone receptor regulation [69]. The rs10133012 locus is proximal to the EIF5 gene, associated with protein translation initiation, potentially affecting the expression of key enzymes regulating cholesterol metabolism [70]. The rs117154811 locus resides in the PCSK5 and RFK gene region, which are involved in proprotein conversion and vitamin B2 metabolism, closely related to lipid metabolism [71, 72]. Importantly, rs118123321 is near the NDUFC2 and GAB2 genes, which participate in mitochondrial respiratory chain function and growth factor receptor signal transduction, respectively, suggesting that 7‐HOCA may influence NAFLD development through effects on energy metabolism and cellular signalling pathways [73]. The rs75165358 locus is adjacent to the ADAM33 and SIGLEC1 genes, which are associated with inflammatory responses and immune regulation, indicating that 7‐HOCA might influence NAFLD pathological progression through modulation of inflammatory processes [74, 75]. Collectively, these genetic variants suggest that metabolic regulation in the brain‐liver axis may be partially controlled by a shared genetic network, providing a key to understanding the metabolic dialogue between the CNS and the liver.

As a key intermediate in cholesterol metabolism, 7‐HOCA is primarily produced by neurons and astrocytes in the brain and participates in regulating neuronal cholesterol homeostasis [15, 76]. The CNS's sensitivity to cholesterol metabolism far exceeds that of peripheral organs, primarily due to three key factors [77, 78]: the brain contains approximately 25% of the body's cholesterol despite constituting only 2% of body weight; the blood–brain barrier strictly limits cholesterol exchange between central and peripheral compartments; and neurons and glial cells express specific cholesterol receptors and transporters that exhibit heightened sensitivity to changes in cholesterol levels. Consequently, 7‐HOCA levels in CSF can more sensitively and earlier reflect systemic lipid metabolism dysregulation.

When 7‐HOCA from CSF is released into the systemic circulation across the blood–brain barrier, it can directly influence the liver's lipid processing capacity. Specifically, 7‐HOCA may promote hepatic fatty acid synthesis and lipid accumulation by activating the liver X receptor signalling pathway, while simultaneously inhibiting the activity of CYP7A1, a key enzyme in bile acid synthesis, leading to cholesterol metabolism dysregulation [79, 80]. Due to the CNS's high sensitivity to cholesterol metabolism alterations, changes in CSF 7‐HOCA levels may precede serum indicators, providing an early warning signal for lipid metabolism disorders.

In contrast, Uracil, a product of pyrimidine nucleotide metabolism, primarily originates from nucleic acid metabolism in neurons and glial cells in the CSF. We found Uracil to be negatively associated with NAFLD, likely because Uracil participates in several key protective pathways: promoting hepatic fatty acid oxidation by regulating the AMPK signalling pathway; enhancing hepatocyte mitochondrial function and oxidative stress defence systems as a critical precursor for RNA repair; and modulating hepatic inflammatory responses through the neuro‐endocrine‐immune network [81, 82]. In summary, these findings reveal a complex brain‐liver metabolic communication network where CSF metabolites may influence hepatic health through multiple pathways: directly acting on the liver after crossing the blood‐brain barrier; regulating hepatic metabolism via neural pathways such as the vagus nerve; and modulating systemic metabolic status by affecting the hypothalamic‐pituitary axis. This multi‐level regulatory mechanism explains why metabolic changes in the CNSmay represent upstream regulators of NAFLD development rather than merely secondary responses to the disease.

In this study, we utilised the MR method, a robust statistical tool that uses genetic variations as IVs to estimate causal relationships between exposures and outcomes. The main advantage of the MR method is its ability to enhance the reliability of causal inferences by reducing the interference of confounding factors common in conventional observational studies, such as lifestyle and environmental factors [83, 84]. Additionally, the MR method is less susceptible to reverse causation and confounding variables, providing a clearer and more reliable understanding of the relationship between CSF metabolites and NAFLD. However, despite the many advantages of the MR method, the study has some limitations. First, the genetic instruments used in this study explain only a small proportion of the variance in CSF metabolite levels. This limitation is common in metabolomics MR studies due to the complex genetic architecture of metabolite concentrations, which may affect the statistical power to detect causal relationships. Additionally, our study was conducted primarily in populations of European ancestry, which limits the generalisability of our findings to other ethnic groups. The genetic variants associated with metabolite levels may differ across populations due to linkage disequilibrium patterns and allele frequency differences, potentially affecting the validity of our results in non‐European populations.

Second, our MR analysis faced specific challenges when dealing with binary variables. When binary outcomes (such as NAFLD status) are involved in MR analysis, there are methodological limitations in interpreting the causal estimates. The non‐collapsibility of OR in non‐linear models may lead to biased estimates, and the binary nature of the outcome can reduce statistical power compared to continuous outcomes. Similarly, when binary exposures are analysed, the instrumental variable assumptions become more difficult to satisfy, potentially leading to weak instrument bias and violating the exclusion restriction assumption.

Third, the accuracy of metabolite detection is crucial for the study results. If there are measurement errors in the metabolites, it may lead to biased causal estimates. Moreover, although our sample size is relatively large, providing some statistical power, the sample's racial and geographical distribution is limited to specific regions, potentially restricting the generalisability of the study results.

Fourth, we acknowledge the invasive nature of CSF sample collection as a major practical limitation for translating these biomarkers into widespread clinical applications. Compared to non‐invasive diagnostic approaches for NAFLD (such as ultrasound) or even other invasive methods (like liver biopsy), lumbar puncture for CSF collection carries its own procedural risks and patient acceptability challenges. This limits the feasibility of using CSF metabolites as routine diagnostic biomarkers for NAFLD screening in general populations. Future studies should explore the expression of these metabolic pathways in more accessible sample types, such as blood or urine, which would substantially improve clinical applicability.

Given the potential roles of the metabolites identified in this study in NAFLD pathogenesis, we propose a phased approach for future research. First, immediate validation could be performed by examining associations between these CSF metabolites and established NAFLD markers such as ALT, AST and imaging‐based assessments of hepatic steatosis within existing datasets. This approach would provide direct validation of our MR findings using conventional liver function indicators. Second, validation studies using independent cohorts and alternative methodological approaches are needed to confirm our findings across different populations. Third, mechanistic studies exploring the biological pathways through which these metabolites influence NAFLD development would deepen our understanding of disease pathogenesis. Following these confirmatory steps, translational research could evaluate whether similar metabolic patterns are detectable in more accessible samples such as blood or urine, potentially leading to practical biomarker development. Only after substantial validation should interventional studies be considered to assess whether modulating these metabolite pathways affects NAFLD outcomes. Ultimately, with sufficient supporting evidence, larger clinical trials and multicenter studies would be warranted to fully evaluate clinical applications.

## 5 Conclusion

This study, through MR analysis, has revealed causal relationships between specific CSF metabolites and NAFLD, particularly highlighting the levels of 7‐HOCA and Uracil. The discovery of these metabolites emphasises the significant role of the brain‐liver axis in metabolic regulation and the potential connection between the CNS and liver health. These findings provide new perspectives for understanding the deeper pathological mechanisms of NAFLD and offer a scientific foundation for developing targeted therapeutic strategies and preventive measures.

## Author Contributions

**Fang Liang:** data curation (equal), investigation (equal), resources (equal), visualization (equal), writing – original draft (equal), writing – review and editing (equal). **Tiegang Xiao:** formal analysis (equal), software (equal), writing – review and editing (equal). **Bing Wang:** conceptualization (equal), project administration (equal), writing – review and editing (equal).

## Ethics Statement

We employed publicly available GWAS summary statistics data obtained from the OpenGWAS database, which collects data from studies conducted with appropriate informed consent protocols approved by institutional review boards. As a result, our study does not require a separate ethics statement.

## Consent

The authors have nothing to report.

## Conflicts of Interest

The authors declare no conflicts of interest.

## Supporting information


**Figure S1:** Forest plot for the causal effect of indivibual SNPs in discovery group.

**Figure S2:** Forest plot for the causal effect of indivibual SNPs in validation group.

**Figure S3:** Forest plot for the results of leave‐one‐out analysis in discovery group.

**Figure S4:** Forest plot for the results of leave‐one‐out analysis in validation group.

**Table S1:** SNPs related to metabolite levels in cerebrospinal fluid in discovery group.

**Table S2:** SNPs related to metabolite levels in cerebrospinal fluid in validation group.

**Table S3:** The MR results between cerebrospinal fluid metabolites and NAFLD in the discovery group.

**Table S4:** The MR results between cerebrospinal fluid metabolites and NAFLD in the validation group.

**Table S5:** heterogeneity and pleiotropy results for all directions.

**Table S6:** NAFLD‐associated SNPs for inverse MR analysis in discovery cohort.

**Table S7:** NAFLD‐associated SNPs for inverse MR analysis in validation cohort.

**Table S8:** The results of inverse Mendelian randomisation analysis in discovery group.

**Table S9:** The results of inverse Mendelian randomisation analysis in validation group.

## Data Availability

The datasets generated and/or analysed during the current study are available in the IEU open GWAS project (https://gwas.mrcieu.ac.uk/).
