# Supplementary figures and images for: The Role of Metabolites in CSF on NAFLD Development: A Mendelian Randomisation Analysis
Source: Endocrinol Diabetes Metab. 2025 Nov 23;8(6):e70088. doi: 10.1002/edm2.70088 (PMC12640884; doi:10.1002/edm2.70088)

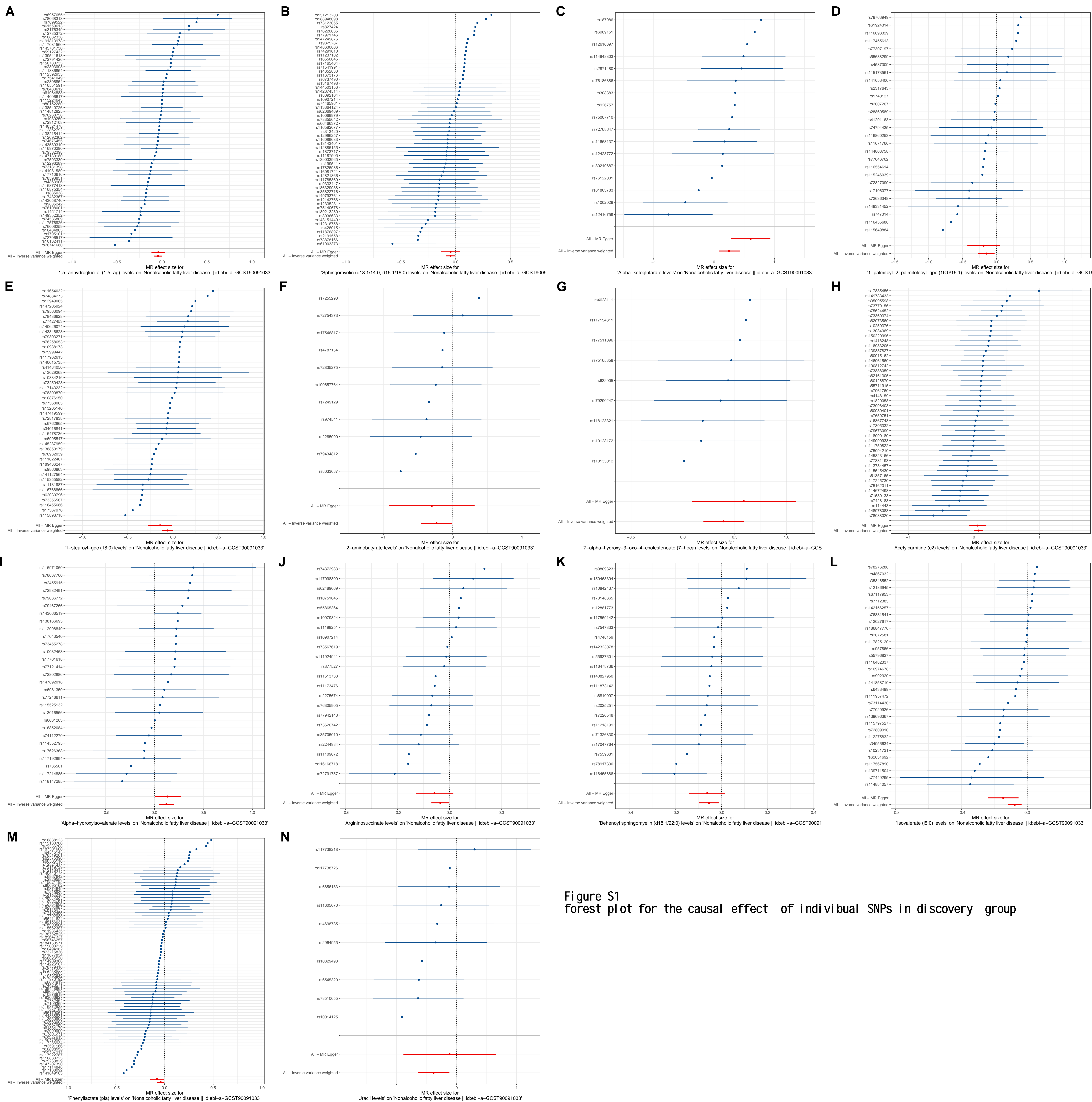

Supplement: Supplementary file 1 — Figure S1: Forest plot for the causal effect of indivibual SNPs in discovery group. [file EDM2-8-e70088-s013.pdf]

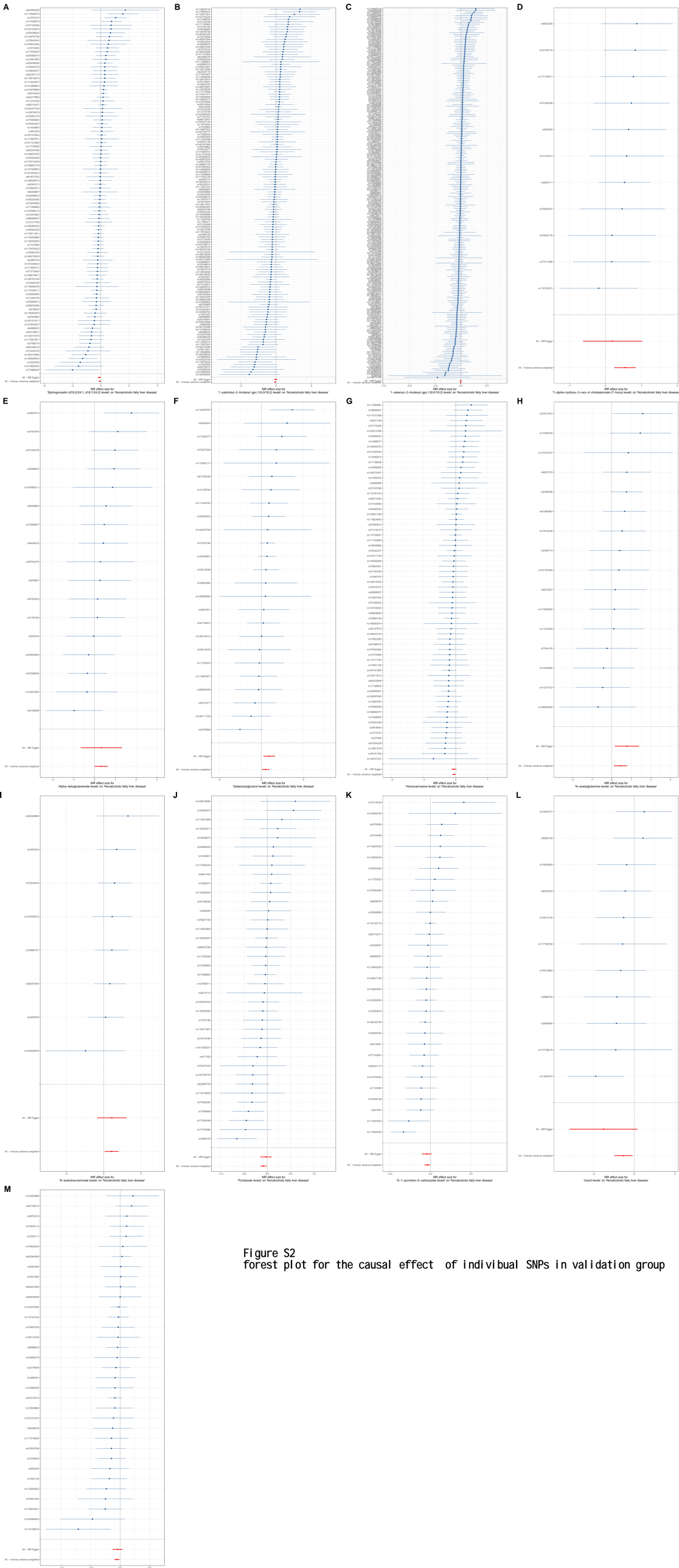

Supplement: Supplementary file 2 — Figure S2: Forest plot for the causal effect of indivibual SNPs in validation group. [file EDM2-8-e70088-s012.pdf]

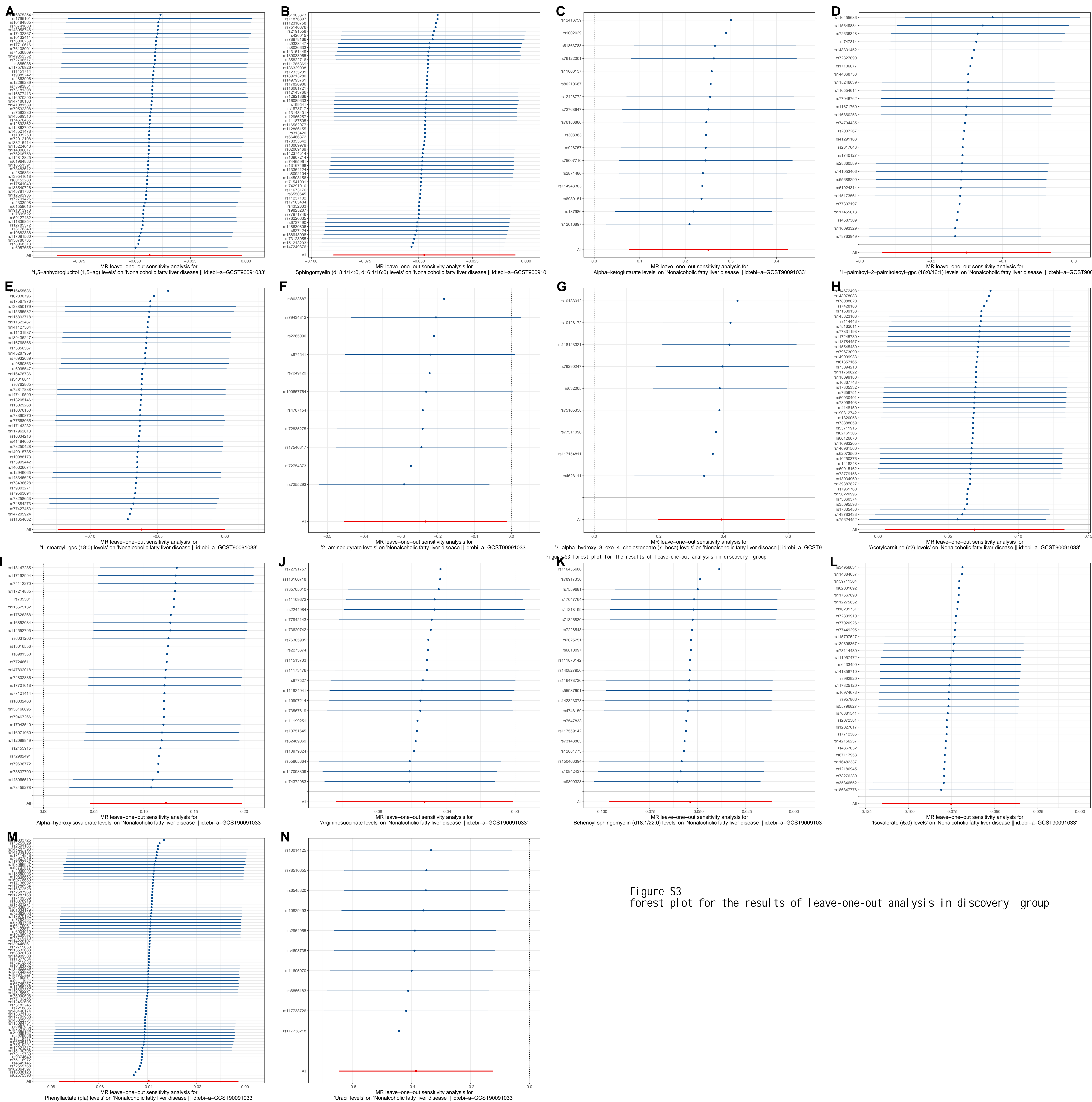

Supplement: Supplementary file 3 — Figure S3: Forest plot for the results of leave‐one‐out analysis in discovery group. [file EDM2-8-e70088-s001.pdf]

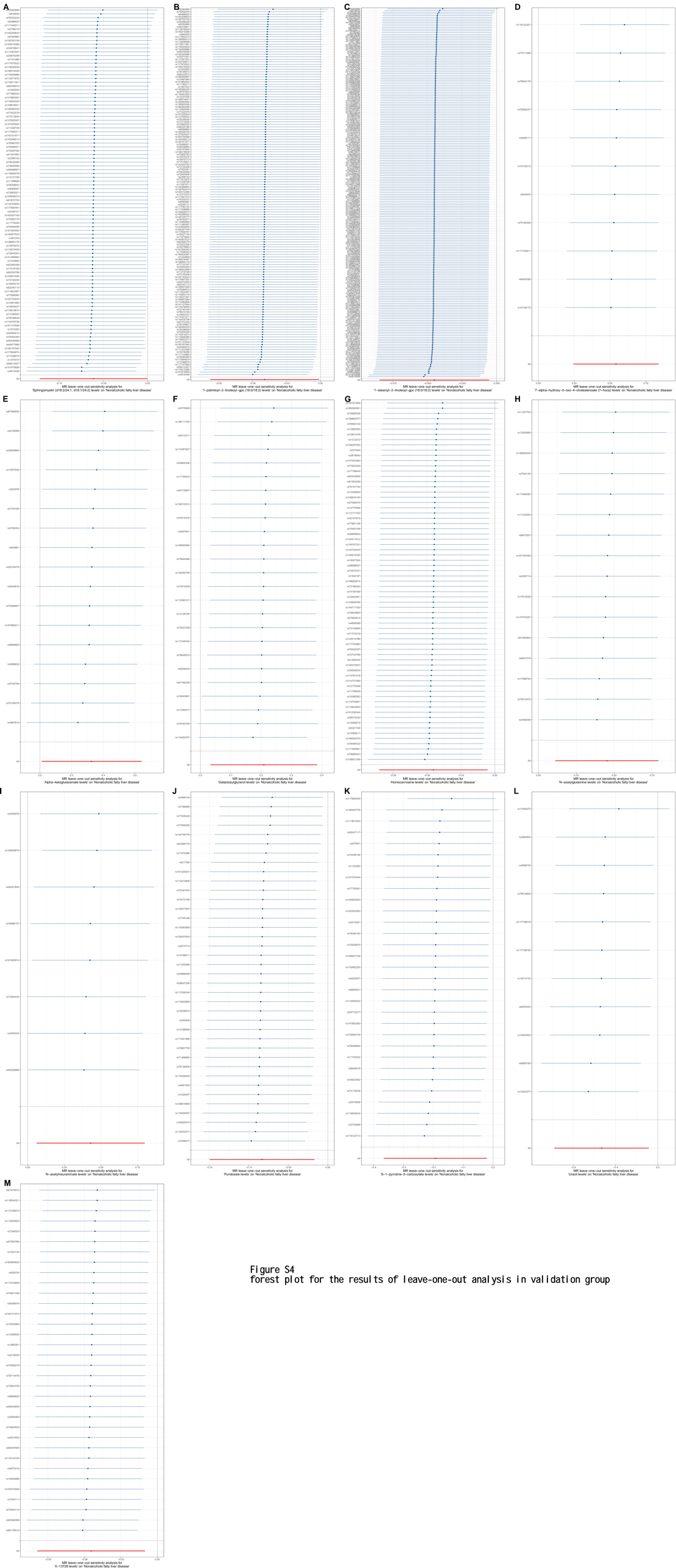

Supplement: Supplementary file 4 — Figure S4: Forest plot for the results of leave‐one‐out analysis in validation group. [file EDM2-8-e70088-s006.pdf]
